# Non-Invasive Assessment of Hypoxia and Neovascularization with MRI for Identification of Aggressive Breast Cancer

**DOI:** 10.3390/cancers12082024

**Published:** 2020-07-24

**Authors:** Barbara Bennani-Baiti, Katja Pinker, Max Zimmermann, Thomas H. Helbich, Pascal A. Baltzer, Paola Clauser, Panagiotis Kapetas, Zsuzsanna Bago-Horvath, Andreas Stadlbauer

**Affiliations:** 1 Department of Biomedical Imaging and Image-Guided Therapy, Division of Molecular and Gender Imaging, Medical University of Vienna, 1090 Vienna, Austria; barbara.bennani-baiti@meduniwien.ac.at (B.B.-B.); katja.pinker@meduniwien.ac.at (K.P.); pascal.baltzer@meduniwien.ac.at (P.A.B.); paola.clauser@meduniwien.ac.at (P.C.); panagiotis.kapetas@meduniwien.ac.at (P.K.);; 2 Department of Radiology, Breast Imaging Service, Memorial Sloan Kettering Cancer Center, New York, NY 10065, USA; 3 Department of Neurosurgery, University of Erlangen-Nürnberg, 91054 Erlangen, Germany; Max.Zimmermann@med.uni-tuebingen.de (M.Z.); andi@nmr.at (A.S.); 4 Department of Preclinical Imaging and Radiopharmacy, University of Tübingen, 72076 Tübingen, Germany; 5 Department of Pathology, Medical University of Vienna, 1090 Vienna, Austria; zsuzsanna.bago-horvath@meduniwien.ac.at; 6 Institute of Medical Radiology, University Clinic of St. Pölten, 3100 St. Pölten, Austria

**Keywords:** breast cancer, hypoxia, neovascularization, magnetic resonance imaging, tumor aggressiveness

## Abstract

The aim of this study was to investigate the potential of magnetic resonance imaging (MRI) for a non-invasive synergistic assessment of tumor microenvironment (TME) hypoxia and induced neovascularization for the identification of aggressive breast cancer. Fifty-three female patients with breast cancer underwent multiparametric breast MRI including quantitative blood-oxygen-level-dependent (qBOLD) imaging for hypoxia and vascular architecture mapping for neovascularization. Quantitative MRI biomarker maps of oxygen extraction fraction (OEF), metabolic rate of oxygen (MRO2), mitochondrial oxygen tension (mitoPO2), microvessel radius (VSI), microvessel density (MVD), and microvessel type indicator (MTI) were calculated. Histopathology was the standard of reference. Histopathological markers (vascular endothelial growth factor receptor 1 (FLT1), podoplanin, hypoxia-inducible factor 1-alpha (HIF-1alpha), carbonic anhydrase 9 (CA IX), vascular endothelial growth factor C (VEGF-C)) were used to confirm imaging biomarker findings. Univariate and multivariate regression analyses were performed to differentiate less aggressive luminal from aggressive non-luminal (HER2-positive, triple negative) malignancies and assess the interplay between hypoxia and neoangiogenesis markers. Aggressive non-luminal cancers (*n* = 40) presented with significantly higher MRO2 (i.e., oxygen consumption), lower mitoPO2 values (i.e., hypoxia), lower MTI, and higher MVD than less aggressive cancers (*n* = 13). Data suggest that a model derived from OEF, mitoPO2, and MVD can predict tumor proliferation rate. This novel MRI approach, which can be easily implemented in routine breast MRI exams, aids in the non-invasive identification of aggressive breast cancer.

## 1. Introduction

Breast cancer is characterized by considerable heterogeneity, resulting in varying genetic, phenotypic and behavioral characteristics, clinical presentations, and treatment responses [1,2,3,4,5,6]. This recognized tumor heterogeneity and the lack of understanding thereof significantly contributes to treatment failures and patient deaths [7,8]. To date, risk stratification and clinical decision-making in breast cancer have focused on molecular biology and cancer genomics. Yet the focus on tumor molecular profiles ignores the fact that tumor growth and progression does not only depend on the tumor cells’ molecular toolbox, but also on the tumor microenvironment (TME), i.e., features of the tissues that host the cancer and their interaction with the tumor. The TME has been identified as a critical factor of cancer development and progression and current concepts favor that tumor heterogeneity is driven by the combined effect of genomic instability and differential selective pressures from the TME [9]. 

In breast cancer, TME hypoxia and the induced neovascularization have been recognized as key drivers of the development of an aggressive and treatment-resistant tumor phenotype and are strong prognostic factors for disease progression, metastases, and survival [8,10]. In addition, several studies have reported that microvessel density (MVD) is associated with poorer recurrence-free, cancer-specific, and overall survival [11,12,13] as well as with clinical response to chemotherapy [14]. Therefore, an approach that enables a non-invasive synergistic assessment of hypoxia and neovascularization for the identification of aggressive breast cancer could provide actionable information for clinical decision-making.

We have recently developed a novel MRI approach for the non-invasive assessment of hypoxia and neovascularization in benign and malignant breast tumors, which can be easily integrated into a clinical MRI protocol, requiring less than seven minutes of additional scan time and no additional injection of gadolinium-based contrast agents (GBCAs) [15]. In our pilot study, we reported that the proposed approach showed potential to improve tumor characterization and can provide insight into the intratumoral heterogeneity of breast tumors [15]. 

The aim of this study was to investigate the potential of this non-invasive synergistic assessment of TME hypoxia and induced neovascularization for the differentiation of more aggressive and less aggressive breast cancer.

## 2. Materials and Methods 

All subjects gave their informed consent for inclusion before they participated in the study. The study was conducted in accordance with the Declaration of Helsinki, and the protocol was approved by the Ethics Committee of the Medical University of Vienna (Project identification code 297/2007).

### 2.1. Patients

Between October 2016 and July 2017, 76 patients were consecutively included and underwent multiparametric contrast-enhanced MRI of the breast for the assessment of an imaging abnormality detected at conventional breast imaging. All patients met the following inclusion criteria: 18 years or older, not pregnant or breastfeeding at the time of the examination, suspicious imaging finding at mammography or breast ultrasound (Breast Imaging Reporting and Data System [BI-RADS] assessment category 4–5) [16], no previous treatment (i.e., breast biopsy before MRI, neoadjuvant chemotherapy), and no contraindications to MRI or MR contrast agents; 53 were subsequently diagnosed with invasive breast cancer as the study population. The median age of this population was 57 years (range: 34–90 years). Some patients have been previously analyzed and reported in our pilot study [15].

### 2.2. MRI Data Acquisition, Processing, and Biomarker Calculation

All patients underwent MRI exams on a 3 Tesla scanner (Tim Trio, Siemens, Erlangen, Germany) equipped with a standard 16-channel breast coil (Sentinelle, Invivo, Gainesville, FL, USA). The standard MRI protocol comprised of three sequences: an axial T2-weighted turbo spin echo sequence (TR/TE: 4630/194 ms, in-plane resolution: 0.5 × 0.5 mm, slice thickness: 2.5 mm, number of slices: 33); a single-shot diffusion-weighted echo-planar imaging sequence (TR/TE: 6000/66 ms, in-plane resolution: 1.1 × 1.1 mm, slice thickness: 4 mm, number of slices: 33 slices, b-values: 0 and 850 s/mm^2^), and a 3-dimensional T1-weighted fast low-angle shot sequence (TR/TE: 4.0/1.4 ms; in-plane resolution: 0.9 × 0.9 mm; slice thickness: 0.9 mm; 160 slices) which was performed once before and 5 times after contrast injection (gadoterate meglumine, 0.1 mmol/kg body weight) for dynamic contrast-enhanced (DCE) perfusion imaging.

In addition to the standard MRI protocol, we performed quantitative blood-oxygen-level-dependent (qBOLD) imaging, comprising a multi-echo gradient echo sequence (8 echoes; TR: 750 ms, TE: 5–40 ms) for T2*-mapping and a multi-echo spin echo sequence (8 echoes; TR: 2000 ms; TE: 15–120 ms) for T2-mapping. We also performed vascular architecture mapping (VAM), comprising a diffusion-weighted imaging sequence (b-values: 0 and 850 s/mm2; TR/TE: 3000/53 ms; TA 50 s) and a dynamic susceptibility contrast (DSC) bolus-tracking perfusion sequence combined with a hybrid single-shot gradient echo (GE) spin echo–echo planar imaging (EPI) readout [17] (TR: 1360 ms; TE for gradient echo (GE): 25 ms; TE for spin echo (SE): 93 ms). Identical geometric parameters were applied to all qBOLD and VAM sequences (coronal slice orientation; field-of-view: 320 × 240 mm; in-plane resolution: 2.5 × 2.5 mm, slice thickness: 6 mm; 8 slices). DSC gradient echo spin echo (GESE) perfusion yielded 60 dynamic volumes of both GE-EPI and SE-EPI for tracking the first-pass peak contrast media bolus dynamics. 

Processing of qBOLD and VAM data as well as calculation of MRI biomarker maps for oxygen metabolism and neovascularization were performed with custom-made MATLAB (MathWorks, Natick, MA, USA) software. qBOLD data processing yielded MRI biomarker maps of oxygen metabolism, including oxygen extraction fraction (OEF; percent of the oxygen removed from the blood for tissue consumption), metabolic rate of oxygen (MRO2; rate of oxygen consumed by the tissue in µmol/100 g per min.), and average mitochondrial oxygen tension (mitoPO2; balance between the delivery and consumption of oxygen, i.e., tissue oxygen tension). VAM data processing yielded MRI biomarker maps of neovascularization, including MVD, vessel size index (VSI, i.e., microvessel radius), and microvessel type indicator (MTI) (Table 1). Further details regarding imaging acquisition and processing have been previously reported [15].

### 2.3. Quantitative Data Extraction

Two-dimensional regions of interest (2D ROIs) were manually defined by one reader (SA) based on features seen on the MVD maps for high, medium, and low neovascularization in order to investigate the intratumoral spatial heterogeneity of neovascularization, oxygen metabolism, and hypoxia. MVD were chosen for two reasons: (i) MVD is a common parameter for neovascularization, and (ii) it was demonstrated in a previous study that MVD is best suited for detection of neovascularization in brain tumors. Cut-off values were obtained from previous studies [18].

MRI biomarker values for oxygen metabolism (OEF, MRO2, mitoPO2; Table 1) and for neovascularization (MVD, VSI, MTI; Table 1) were then averaged for each ROI. Total tumor volumes were calculated based on tumor extent on DCE-MR images. The ellipsoid volume formula V = π/6 × L × W × H was used for the calculation of tumor volumes.

### 2.4. Histopathological Reference Standard

Diagnosis was established by an experienced specialized breast pathologist (ZBH). All lesions were histopathologically verified by image-guided needle biopsy or surgery. For all invasive breast cancers, histopathology results were reviewed for tumor subtype according to world health organisation (WHO) classification, and tumor stage and grade according to Elston and Ellis [19]. Breast cancer intrinsic subtype was determined by immunohistochemistry based on estrogen receptor (ER), progesterone receptor (PR), and human epidermal growth receptor 2 (HER2) status as well as Ki67 expression according to current guidelines [20] and defined as luminal A, luminal B, HER2-positive, and triple negative (TN) [21,22]. Patients with equivocal HER2 status were evaluated using chromogenic in situ hybridization to detect gene amplification. HER2-positive and TN breast cancers were considered more aggressive breast cancers with a worse prognosis than luminal A/B breast cancers.

Hypoxia-inducible factor 1-alpha (HIF-1alpha), carbonic anhydrase 9 (CA IX), vascular endothelial growth factor C (VEGF-C), vascular endothelial growth factor receptor 1 (FLT1), and Podoplanin (all Ventana, Tucson, AR, USA) staining were performed on selected specimens, depending on the availability of the specimens. Immunohistochemistry staining was performed using an automated Ventana Benchmark Ultra (Ventana, Tucson, AR, USA) staining device.

### 2.5. Statistical Analysis

Nominal data were presented using absolute frequencies and percentages. Metric data were presented using means ± SD if normally distributed or median (min, max) if skewed. SPSS 25.0 (IBM Corp., Armonk, NY, USA) and R statistics (R Foundation, Vienna, Austria) were used for statistical evaluation. Unpaired *t*-tests were performed to compare median MRI-derived imaging metrics and to differentiate “less aggressive” luminal (luminal A, luminal B) from “more aggressive” non-luminal (HER2-postive, TN) malignancies. The association between qBOLD and VAM markers, PR, ER, p53, ki67 and tumor size was explored by univariate Spearman rank correlation coefficient calculation and multivariable linear regression analysis using backward feature selection (entry and remove limits: *p* < 0.05 and *p* < 0.1). Variance Inflation Factors (VIFs) were calculated to identify collinearity, considering VIFs below 3 as acceptable and values > 10 as definitely inacceptable. *p* values less than 0.05 were deemed statistically significant. 

## 3. Results

There were 53 malignant breast tumors (mean tumor volume: 6.54 mL) in 53 patients. There were 40 (75.5%) less aggressive luminal and 13 (24.5%) more aggressive non-luminal breast cancers. Table 2 illustrates the patients’ clinical and histopathological information. MRI measurement of TME hypoxia and neovascularity with qBOLD and VAM was successfully performed in all breast cancers. 

### 3.1. Identification of Aggressive Breast Cancer

**Figure 1:**
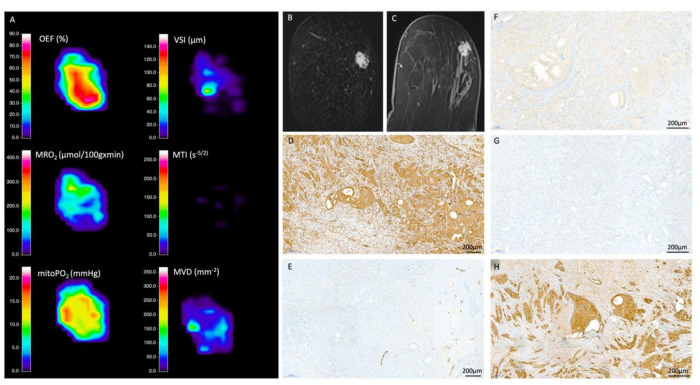
Radiologic-histopathologic correlation: 58-year-old patient with an invasive ductal carcinoma (estrogen receptor (ER)/ progesterone receptor (PR) positive and human epidermal growth receptor 2 (HER2) negative, ki-67 20%). (**A**) MRI biomarkers (**B**) Contrast enhanced T1 (CE-T1) image coronal (**C**) CE-T1 image axial (**D**) vascular endothelial growth factor receptor 1 (FLT1) (**E**) podoplanin (**F**) hypoxia-inducible factor 1-alpha (HIF-1alpha) (**G**) carbonic anhydrase 9 (CA IX) (**H**) vascular endothelial growth factor C (VEGF-C). HIF-1alpha is expressed ubiquitously, along with VEGF-C and FLT1 in the more solid areas of the tumor. This is matched by higher mitoPO2 values and a higher OEF (especially in the tumor center) and lower MRO2 levels compared to the more aggressive triple negative tumor. Concordantly, CA IX is not expressed. MVD is lower as compared with the more aggressive lesion in Figure 2.

**Figure 2:**
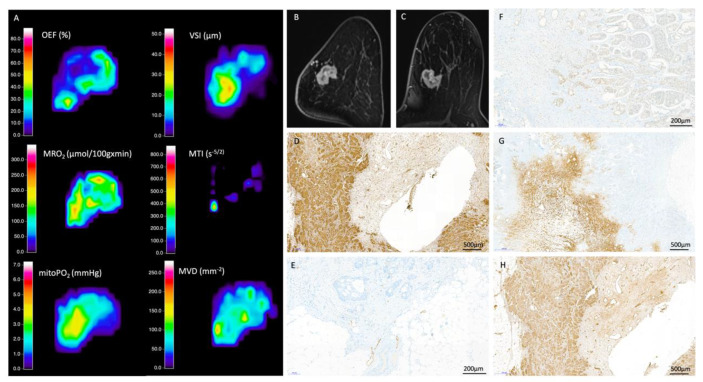
Radiologic-histopathologic correlation: 83-year-old patient with an invasive ductal carcinoma (ER/PR−, HER2−, ki-67 30%). (**A**) MRI biomarkers (**B**) CE-T1 image coronal (**C**) CE-T1 image axial (**D**) FLT 1 (**E**) Podoplanin (**F**) HIF-1alpha (**G**) CA IX H VEGF-C. (**H**) VEGF-C. CE-T1 displays a tumor with a fibrotic center and rim enhancement. The blank center represents the biopsy clip region. The fibrotic center shows strong CA IX staining, indicating severe hypoxia corresponding to the MRI hypoxia imaging marker findings. HIF-1alpha, VEGF-C, and FLT1 staining are significantly stronger in this exemplary non-luminal tumor as opposed to Figure 1. They verify the hypoxia imaging markers. An arterial feeding vessel is seen on the left lower perimeter of the lesion that is reflected by a bright yellow signal on the left lower perimeter of the lesion in the MTI image. The purple hues of the MTI imaging represent venous draining vessels, matched by histopathological staining results. Venous draining vessels are more predominant in more aggressive lesions as illustrated in this example of a TN.

From QBOLD mapping, MRO2 was significantly lower in less aggressive luminal (129.4 ± 19.7 µmol/100 g·min) compared to aggressive non-luminal breast cancers (146.9 ± 19.1 µmol/100 g·min; *p* = 0.007). The opposite was true for mitoPO2 that was significantly higher in less aggressive luminal (13.3 ± 7.1 mmHg) compared to aggressive non-luminal breast cancers (8.6 ± 4.1 mmHg; *p* = 0.006) (Table 3). Hypoxia correlated with the proliferation rate and biologic aggressiveness of breast cancer as indicated by immunohistochemistry expression and distribution of common markers of hypoxia (HIF-1alpha, VEGF-C, CA IX, and FLT-1). Figure 1 and Figure 2 exemplify CA IX staining in in a luminal B tumor and a non-luminal triple negative tumor, respectively. CA IX, a marker that is expressed only upon severe hypoxia, was strongly expressed in the fibrotic center of the TN tumor but not the luminal B tumor, representing the lower and higher mitoPO2 values seen on MRI, respectively. 

**Figure 3:**
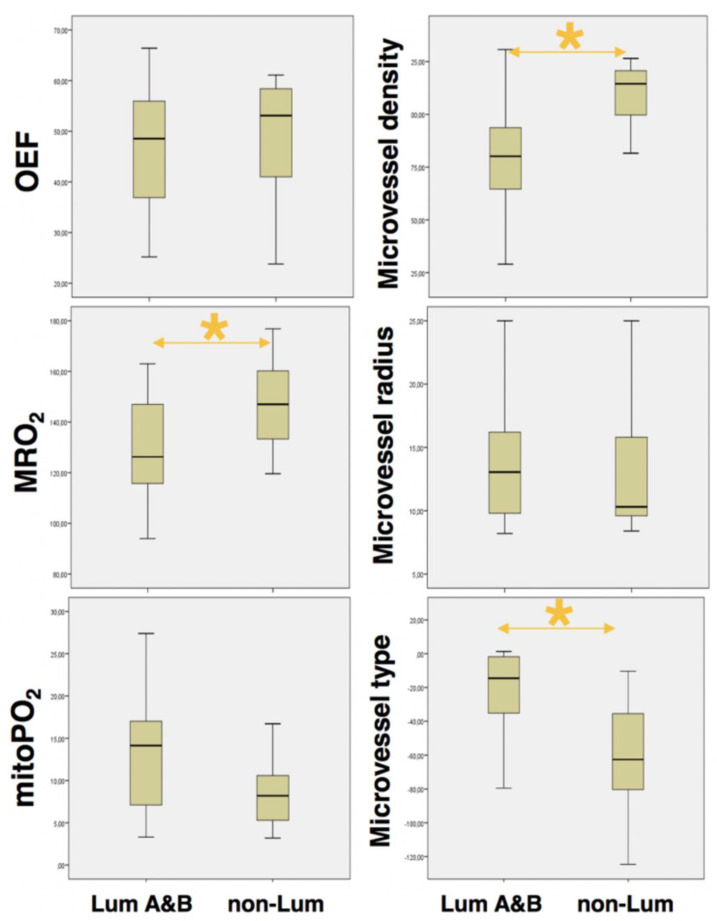
Box and whisker plots show the imaging biomarkers for luminal (A and B) and non-luminal (HER-2 positive and TN) tumors. Boxes are mean values ± standard deviations, and whiskers indicate minimum and maximum values. * indicates statistical significance (*p* < 0.05).

From VAM mapping, aggressive non-luminal cancers had a significantly higher MVD than less aggressive luminal cancers (110.6 ± 14.6 mm^−2^ vs. 81 ± 24.3 mm^−2^; *p* < 0.001). In contrast, aggressive non-luminal cancers had lower MTI than less aggressive luminal cancers (−62.1 ± 39.2 s^−5/2^ vs. −22.4 ± 25.6 s^−5/2^, *p* < 0.001), showing that aggressive non-luminal cancers had a microvessel type that was significantly more tortuous with slow flowing blood (capillary/venous) (Figure 1, Figure 2 and Figure 3, Table 3). The increase in MVD was reflected by an increase in tumor vasculature as illustrated in the histopathological sample cases.

### 3.2. QBOLD Mapping and Histopathological Markers

**Figure 4:**
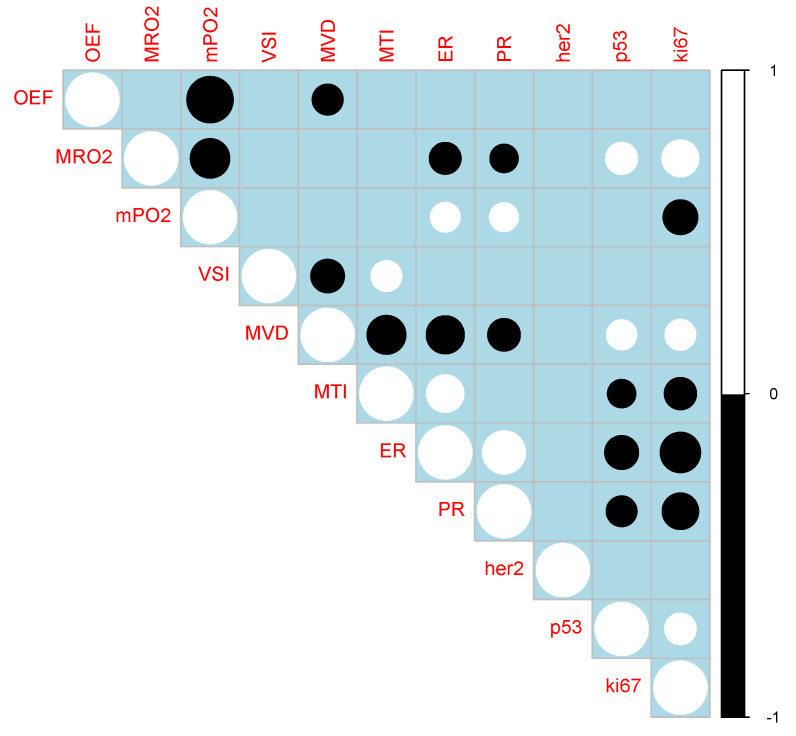
Correlation matrix of Spearman’s rank correlation coefficients. White indicates positive, whereas black indicates negative correlations. Size corresponds to strength of correlation. All fields with an entry were statistically significant (*p* < 0.05).

Univariate analysis (Figure 4) showed a negative correlation between MRO2 and ER as well as PR expression, along with a positive correlation between MRO2 and p53 and ki67 (*p* < 0.05). mitoPO2 positively correlated with ER and PR expression and negatively correlated with ki67 (*p* < 0.05). No statistically significant correlation was found for QBOLD derived indicators and HER2 expression or lesion size (*p* > 0.05). OEF and lesion volume did not display a statistically significant correlation with any of the investigated histopathological markers (*p* > 0.05).

### 3.3. VAM Mapping and Histopathological Markers

Univariate analysis (Figure 4) showed a negative correlation between MVD and ER and PR expression and a positive correlation between MVD and p53 as well as ki67 (*p* < 0.05). MTI positively correlated with ER expression and negatively correlated with p53 and ki67 (*p* < 0.05). No statistically significant correlation was found for VAM derived indicators and HER2 expression or lesion size (*p* > 0.05). VSI and lesion volume did not display a statistically significant correlation with any of the investigated histopathological markers (*p* > 0.05).

### 3.4. Prediction of ki67 by QBOLD and VAM Imaging

**Figure 5:**
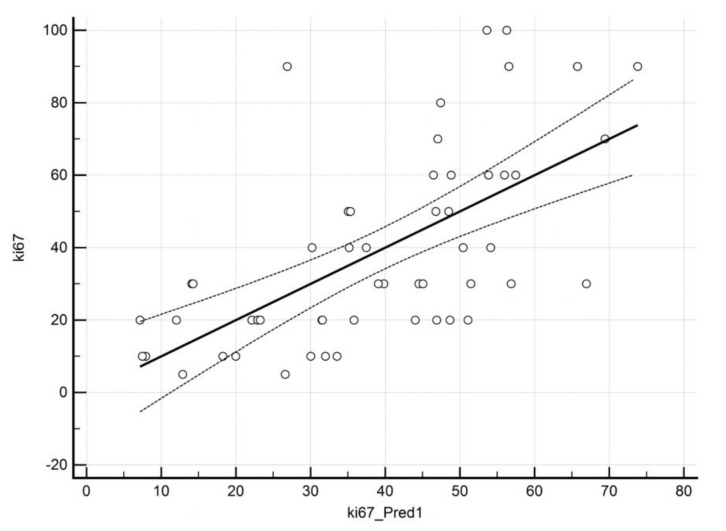
Scatterplot of predicted versus actual ki67 values. Actual ki67 levels (*y* axis) are plotted against predicted ki67 values (*x* axis).

Multiple regression analysis for prediction of ki67 by VAM- and QBOLD-derived markers was based on OEF, mitoPO2, and MVD, with corresponding regression coefficients of −0.07 (standard error, SE: 0.04), *p* = 0.07; −0.29 (SE: 0.07), *p* = 0.0001 and −0.04 (SE: 0.01), *p* = 0.003, respectively. This model yielded an adjusted R^2^ of 0.36 (Table 4). Figure 5 displays a scatterplot of predicted vs actual ki67 values. 

### 3.5. Prediction of Hormone Receptors and p53 by QBOLD and VAM Imaging

Multiple regression analysis for prediction of ER positivity yielded a model based on MRO2, OEF, MVD, and VSI. The overall model exhibited an adjusted R2 of 0.47. In this multivariate model, the following regression coefficients between imaging markers and ER positivity were found: MRO2 −0.06 (SE: 0.02, *p* = 0.004), OEF −0.13 (SE: 0.04, *p* < 0.001), MVD −0.11 (SE: 0.02, *p* < 0.0001), and VSI −0.23 (SE: 0.09, *p* < 0.01) (Table 2).

Multiple regression analysis for prediction of PR positivity yielded a model based on MRO2, OEF, and MVD. The overall model exhibited an adjusted R^2^ of 0.25. In this multivariate model the following regression coefficients between imaging markers and PR positivity were found: MRO2 −0.05 (SE: 0.02, *p* = 0.04), OEF −0.11 (SE: 0.04, *p* < 0.01), and MVD −0.07 (SE: 0.02, *p* < 0.001) (Table 2).

Multiple regression analysis for prediction of p53 expression identified only MVD as an independent predictor with an adjusted R^2^ of 0.298 and a regression coefficient for MVD of 0.04 (SE: 0.02, *p* = 0.01) (Table 2). 

**Figure 6:**
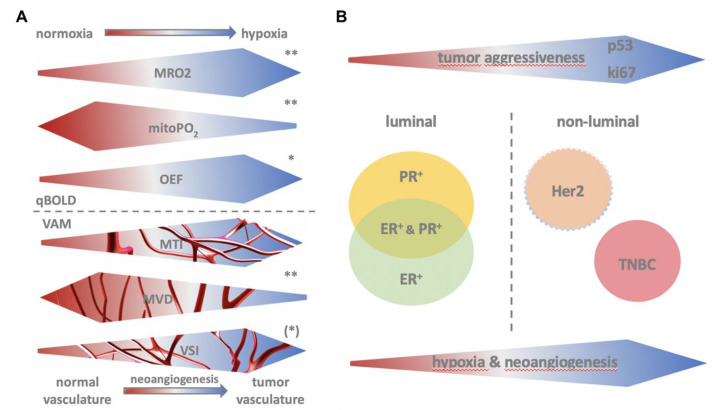
Panel **A**. Illustration of imaging marker behavior in relation to hypoxia and neoangiogenesis. Direction of arrow indicates increase or decrease of values. Asterisks indicate predictive significance as suggested by our data (** highly indicative, * moderately indicative, (*) mildly indicative, schematically based on data derived from Spearman’s rank correlation coefficient analysis). Panel **B**. Schematic illustration of biomarker distribution in relation to hypoxia, neoangiogenesis, and tumor aggressiveness. Triple negative breast cancer (TNBC).

A schematic drawing of the hypothesis upon which this explorative analysis was based is given in Figure 6A,B illustrating the interplay of qBOLD and VAM derived imaging biomarkers of hypoxia and neoangiogenesis with tumor aggressiveness, subtype, and histopathologic markers. 

## 4. Discussion

Recently, we developed a non-invasive synergistic assessment of TME hypoxia and induced neovascularization in breast tumors; our initial results showed that this approach is promising to improve tumor characterization and provide insight into the distinct intratumoral heterogeneity of breast tumors [15]. In this study, we investigated the same algorithm for the differentiation of less aggressive luminal and more aggressive non-luminal breast cancers. In the past, molecular profiling has proven that breast cancer is a disease with a remarkable heterogeneity and that various cancer cell populations co-exist in a given primary tumor that differ significantly in their genetic, phenotypic, and behavioral characteristics. However, breast cancer heterogeneity is not solely driven by the combined effect of genomic instability within the tumor, but also by differential selective pressures from the TME, with hypoxia being one of the most significant TME factors. Hypoxia induces the development of sub-populations of cells within a tumor with an aggressive and treatment-resistant phenotype leading to rapid progression and a poor prognosis [8,23]. To survive and grow in a hostile, hypoxic TME, tumor cells co-opt adaptive mechanisms; one key mechanism is the development of new tumor vessels (“angiogenic switch”) to deliver oxygen and nutrients and remove metabolic waste products [24,25]. 

Our results indicate that the identification of aggressive breast cancer is feasible using our approach. It has to be noted that lesion size did not correlate with indicators of tumor aggressiveness in any of our analyses. A less aggressive slow growing tumor does not undergo the same pathophysiological changes evoked by hypoxia, as does a rapidly dividing, “aggressive” cell population. Morphologic imaging features cannot capture the underlying oncogenic processes, highlighting the importance of quantitative imaging biomarkers of hypoxia and neovascularization in this context. 

In clinical practice, molecular breast cancer subtypes, i.e., luminal A, luminal B, HER2-positive, and TN, are usually derived using immunohistochemistry surrogates to inform on tumor aggressiveness, prognosis, and prediction, and are routinely used to guide recommendations for neo- and adjuvant systemic therapies [20,21]. However, to date, the information on molecular subtypes has to be obtained by invasive tissue sampling, which is a snapshot of a specific tumor region, subject to selection bias and not representative of the tumor in its entirety. Tumor biology is also subject to change over time and with treatment [26]. Therefore, the investigation of novel non-invasive approaches—such as ours—for the identification of aggressive breast cancer that are derived from the tumor in its entirety and be easily repeated over the course of neoadjuvant treatment are warranted. 

Particularly promising, therefore, are the results of our analysis that allow to predict ki67 status through a combined analysis of OEF, mitoPO2, and MVD (Figure 2). Ki67 is a particularly valuable biomarker in breast cancer as higher ki67 indicates higher tumor grade (i.e., increased tumor aggressiveness). It is increased in dividing cell populations and therefore increased in fast growing breast cancers. Accelerated tumor growth implies hypoxia with subsequently induced neoangiogenesis. Therefore, our finding that ki67 is positively linked with MRO2 (i.e., oxygen consumption) and negatively linked with mitoPO2 (tissue oxygen tension) along with increased MVD (mean vascular density) and decreased MTI (i.e., more venous draining vessels) is in agreement with the hypothesis (see Figure 2). 

With regard to tumor aggressiveness, the tumor suppressor p53 is another used biomarker. IHC detection of p53 protein is loosely, but imperfectly, associated with mutations in TP53 [27,28]. An increase of p53 implies a partial/complete loss of p53 function, which contributes to uncontrolled cell growth, ensuing hypoxia, and neoangiogenesis. qBOLD and VAM imaging should therefore also indirectly associate with IHC p53 levels, which we observed both in univariate as well as in multivariate analysis. 

Luminal, less aggressive breast cancer phenotypes that display slower growth, warranting less oxygen supply than more aggressive breast cancer subtypes. We observed higher oxygen tension and lower oxygen consumption, i.e., positive association of mitoPO2 and negative association of MRO2 in luminal breast cancers. VAM mapping derived factors complete this picture, where MVD (i.e., mean vascular density) independently associates with hormone receptor positivity whereas VSI (i.e., vessel size index) negatively associates only with ER positivity in the multivariate analysis.

All of these markers tested separately fit the proposed schematics along which the imaging biomarkers were designed. In the last step of our analysis, we therefore tested whether our approach could accurately predict aggressive tumor subtypes as defined by current histopathological standards.

Specifically, we observed that aggressive non-luminal cancers (HER2 positive and TN) presented with significantly lower MRO2 (i.e., metabolic rate of oxygen) than less aggressive luminal cancers (luminal A and luminal B) and lower mitoPO2 values (i.e., hypoxia) than luminal breast cancers. This reflects the increased oxygen consumption accompanied with increased hypoxia that is exacerbated in fast growing aggressive phenotypes. Moreover, we also observed lower MTI (i.e., microvessel type indicator) and higher MVD (i.e., microvessel density) in non-luminal cancers than in luminal cancers. In our model, we view hypoxia and the consequently induced neoangiogenesis as a complex dynamic process with several feedback loops. As a tumor grows, the increasing cell mass enlarges the space between preexisting blood vessels; thus, the formerly high MVD turns into a low MVD. Simultaneously, this process of rapid cell division warrants higher oxygen supply, and the consequently effected hypoxia induces angiogenesis, which in turn increases MVD with increasing MRO2. 

With respect to this complex dynamic process between hypoxia and neovascularization, one of these factors alone cannot characterize tumor aggressiveness. Our novel MRI approach allows however, a non-invasive simultaneous assessment of hypoxia and neovascularization. While patients with luminal cancer may be offered endocrine therapy in addition to surgery and radiation treatment, and patients with HER2 positive cancer may receive additional targeted treatment with monoclonal antibodies, patients with TN cancer have no currently available targeted treatment [29]. After additional validation, these specific results of our study might have direct clinical consequences, i.e., in preventing the exclusion of patients from adequate therapy when a heterogeneous tumor is present.

In this study, we also performed a direct histopathologic correlation for MRI biomarkers. The pathophysiologic changes depicted using our developed novel MRI approach were shown to be similarly traceable by IHC staining. However, it has to be noted that this analysis relies on the limited specimens from invasive tissue sampling, highlighting the clinical difficulties to comprehensively assess tumor neovascularization and hypoxia in its entirety and to monitor these factors during the course of novel MRI approach neoadjuvant treatment. We observed that hypoxia correlated with the proliferation rate and breast cancer aggressiveness as indicated by immunohistochemistry expression and the distribution of common markers of hypoxia (HIF-1alpha, VEGF-C, CA IX, FLT-1, and MVD). Aggressive tumor subtypes (non-luminal) exhibit higher levels of hypoxia and neovascularization with an increased oxygen consumption compared to slower proliferating luminal phenotypes. In particular, the most aggressive tumors, i.e., TN breast cancer, presented with strong hypoxia only in the fibrotic center as evidenced by CA IX staining. HIF-1alpha localization provided additional support to our imaging data, whereby HIF-1alpha translocated into the nucleus under hypoxic conditions to induce (among others) VEGF expression. In line with this, we observe a predominantly cytoplasmatic distribution in the more intraductal tumor components for both luminal and non-luminal tumors and a stronger nuclear staining in the stromal/invasive parts of the lesions. 

In our initial pilot study [15], we showed that our novel MRI approach provided information for the differentiation of benign and malignant breast. In this study, we expand on its potential to provide an accurate tumor characterization through consistent and direct measurement of neovascularization, oxygen metabolism, and hypoxia. Given the high accuracy of breast cancer detection by routine CE-MRI and the clinical need to obtain biopsy specimen at initial diagnosis, we consider our results to be particularly promising in regard to non-invasive disease monitoring (i.e., during neoadjuvant chemotherapy). While initial classification into luminal and non-luminal cancers will for the foreseeable future still be based on histopathological assessment, the fact that ki67 seems to be highly linked to hypoxia, and neovascularization presents a promising opportunity for qBOLD and VAM derived biomarkers to serve as non-invasive quantitative biomarkers for disease monitoring. 

This study has a few limitations. Due to the lower signal-to-noise ratio of the SE-DSC sequence compared with a standard T1w DCE perfusion sequence, the spatial resolution is limited. Research is ongoing and further improvements of spatial resolution of qBOLD and VAM imaging can be expected and will overcome this current limitation. Due to the high number of simultaneously assessed variables, this study is only exploratory in nature. We therefore cannot exclude, that some data derived from multiple regression analysis are overfit. Larger datasets will be required to establish a clinical algorithm providing actionable information for breast cancer patients by qBOLD and VAM imaging biomarkers and will be the subject of future confirmatory analyses. 

## 5. Conclusions

In conclusion, in this study, we demonstrated that a novel MRI approach for the non-invasive synergistic assessment of TME hypoxia and neovascularization, which can be easily implemented in routine breast MRI exams, enables the non-invasive identification of aggressive breast cancer and may provide diagnostic, prognostic, and predictive indicators derived from the tumor in its entirety to guide treatment decisions. 

## Figures and Tables

**Table 1:** List of acronyms of novel MRI parameters and their according units.

Acronym	Novel MRI Parameters	Unit
qBOLD	quantitative blood-oxygen-level-dependent imaging	
OEF	oxygen extraction fraction	%
MRO2	metabolic rate of oxygen	µmol/100 g × min
mitoPO2	mitochondrial oxygen tension	mmHg
VAM	vascular architecture mapping	
VSI	vessel size index	µm
MVD	microvessel density	mm^−2^
MTI	microvessel type indicator	s^−5/2^

**Table 2:** Clinical and histopathological properties of participants (*n* = 53).

Characteristic	*n* (%)
Entire cohort	53 (100%)
Mean patient age (SD)	57.6 (±13.1y)
Mean tumor volume	6535 mm^3^
Tumor grade	
G1	6 (11%)
G2	27 (51%)
G3	20 (38%)
Luminal A/B	40 (75.5%)
Mean patient age (SD)	53.7 (±14.8y)
Mean tumor volume	3144 mm^3^
Luminal A	9 (17%)
Luminal B	31 (59%)
Non-luminal	13 (24.5%)
Mean patient age (SD)	60 (±11.8y)
Mean tumor volume	16,968 mm^3^
HER2-positive	4 (7.5%)
TN/basal-like	9 (17%)

Abbreviations: G1, grade 1; G2, grade 2; G3, grade 3; HER2; human epidermal growth receptor 2; SD, standard deviation; TN, triple negative.

**Table 3:** MRI biomarkers and *p*-values for non-luminal and luminal malignancies.

MRI Biomarkers (unit)	Tumor Type	Value	Std. Error	*p*-Value
OEF	Non-luminal	48.81	±12.6	0.568
(%)	Luminal A/B	46.55	±12.19	
MRO2 *	Non-luminal	146.93	±19.12	0.007
(µmol/100 g × min)	Luminal A/B	129.45	±19.7	
mitoPO2 *	Non-luminal	8.62	±4.06	0.006
(mmHg)	Luminal A/B	13.3	±7.13	
VSI	Non-luminal	13.81	±6.04	0.966
(µm)	Luminal A/B	13.88	±4.73	
MVD *	Non-luminal	110.56	±14.57	<0.001
(mm^−2^)	Luminal A/B	81	±24.32	
MTI *	Non-luminal	−62.13	±39.27	<0.001
(s^−5/2^)	Luminal A/B	−22.43	±25.6	

Abbreviations: MRO2, metabolic rate of oxygen; MVD, microvessel density; OEF, oxygen extraction fraction; PR, progesterone receptor; QBOLD, quantitative blood-oxygen-level-dependent; VIF, Variance Inflation Factors; VSI, vessel size index, * indicates statistically significant results (*p* < 0.05).

**Table 4:** Multiple regression analysis for prediction of ki67, ER, PR, and p53 positivity by qBOLD and VAM-derived features.

Heading	Coefficient	Std. Error	*p*-Value	VIF	Adjusted R^2^
ki67					0.361
(constant)	74.9				
OEF	−0.072	0.039	0.07	2.55	
mitoPO2	−0.292	0.067	0.0001	2.35	
MVD	0.038	0.012	0.003	1.14	
ER positivity					0.469
(constant)	332.1				
MRO2	−0.056	0.019	0.004	1.03	
OEF	−0.133	0.035	<0.001	1.25	
MVD	−0.109	0.018	<0.0001	1.46	
VSI	−0.235	0.088	0.011	1.31	
PR positivity					0.254
(constant)	218.9				
MRO2	−0.048	0.023	0.038	1.03	
OEF	−0.109	0.041	<0.01	1.13	
MVD	−0.068	0.019	<0.001	1.14	
P53 positivity					0.298
(constant)	−10.6				
MVD	0.038	0.015	0.014	1.0	

Abbreviations: ER, estrogen receptor; MRO2, metabolic rate of oxygen; MVD, microvessel density; OEF, oxygen extraction fraction; PR, progesterone receptor; QBOLD, quantitative blood-oxygen-level-dependent; VAM, vascular architecture mapping; VIF, Variance Inflation Factors; VSI, vessel size index.

## References

[1] Zardavas D., Irrthum A., Swanton C., Piccart M. (2015). Clinical management of breast cancer heterogeneity. Nat. Rev. Clin. Oncol..

[2] Wirapati P., Sotiriou C., Kunkel S., Farmer P., Pradervand S., Haibe-Kains B., Desmedt C., Ignatiadis M., Sengstag T., Schutz F. (2008). Meta-analysis of gene expression profiles in breast cancer: Toward a unified understanding of breast cancer subtyping and prognosis signatures. Breast Cancer Res..

[3] Huber K.E., Carey L.A., Wazer D.E. (2009). Breast cancer molecular subtypes in patients with locally advanced disease: Impact on prognosis, patterns of recurrence, and response to therapy. Semin. Radiat. Oncol..

[4] Haynes B., Sarma A., Nangia-Makker P., Shekhar M.P. (2017). Breast cancer complexity: Implications of intratumoral heterogeneity in clinical management. Cancer Metastasis Rev..

[5] Martelotto L.G., Ng C.K., Piscuoglio S., Weigelt B., Reis-Filho J.S. (2014). Breast cancer intra-tumor heterogeneity. Breast Cancer Res..

[6] Lam S.W., Jimenez C.R., Boven E. (2014). Breast cancer classification by proteomic technologies: Current state of knowledge. Cancer Treat Rev..

[7] Vaupel P. (2008). Hypoxia and aggressive tumor phenotype: Implications for therapy and prognosis. Oncologist.

[8] Ruan K., Song G., Ouyang G. (2009). Role of hypoxia in the hallmarks of human cancer. J. Cell Biochem..

[9] Hanahan D., Weinberg R.A. (2011). Hallmarks of cancer: The next generation. Cell.

[10] Hockel M., Schlenger K., Mitze M., Schaffer U., Vaupel P. (1996). Hypoxia and Radiation Response in Human Tumors. Semin. Radiat. Oncol..

[11] Tsutsui S., Kume M., Era S. (2003). Prognostic value of microvessel density in invasive ductal carcinoma of the breast. Breast Cancer.

[12] Al Murri A.M., Wilson C., Lannigan A., Doughty J.C., Angerson W.J., McArdle C.S., McMillan D.C. (2007). Evaluation of the relationship between the systemic inflammatory response and cancer-specific survival in patients with primary operable breast cancer. Br. J. Cancer.

[13] Bevilacqua P., Barbareschi M., Verderio P., Boracchi P., Caffo O., Dalla Palma P., Meli S., Weidner N., Gasparini G. (1995). Prognostic value of intratumoral microvessel density, a measure of tumor angiogenesis, in node-negative breast carcinoma--results of a multiparametric study. Breast Cancer Res. Treat.

[14] Foekens J.A., Peters H.A., Grebenchtchikov N., Look M.P., Meijer-van Gelder M.E., Geurts-Moespot A., van der Kwast T.H., Sweep C.G., Klijn J.G. (2001). High tumor levels of vascular endothelial growth factor predict poor response to systemic therapy in advanced breast cancer. Cancer Res..

[15] Stadlbauer A., Zimmermann M., Bennani-Baiti B., Helbich T.H., Baltzer P., Clauser P., Kapetas P., Bago-Horvath Z., Pinker K. (2019). Development of a Non-invasive Assessment of Hypoxia and Neovascularization with Magnetic Resonance Imaging in Benign and Malignant Breast Tumors: Initial Results. Mol. Imaging Biol..

[16] D’Orsi C., Sickles E., Mendelson E., Morris E. (2013). ACR BI-RADS^®^ Atlas, Breast Imaging Reporting and Data System.

[17] Xu C., Kiselev V.G., Moller H.E., Fiebach J.B. (2013). Dynamic hysteresis between gradient echo and spin echo attenuations in dynamic susceptibility contrast imaging. Magn. Reson. Med..

[18] Stadlbauer A., Zimmermann M., Kitzwogerer M., Oberndorfer S., Rossler K., Dorfler A., Buchfelder M., Heinz G. (2017). MR Imaging-derived Oxygen Metabolism and Neovascularization Characterization for Grading and IDH Gene Mutation Detection of Gliomas. Radiology.

[19] Elston C.W., Ellis I.O. (1991). Pathological prognostic factors in breast cancer. I. The value of histological grade in breast cancer: Experience from a large study with long-term follow-up. Histopathology.

[20] Vasconcelos I., Hussainzada A., Berger S., Fietze E., Linke J., Siedentopf F., Schoenegg W. (2016). The St. Gallen surrogate classification for breast cancer subtypes successfully predicts tumor presenting features, nodal involvement, recurrence patterns and disease free survival. Breast.

[21] Prat A., Pineda E., Adamo B., Galvan P., Fernandez A., Gaba L., Diez M., Viladot M., Arance A., Munoz M. (2015). Clinical implications of the intrinsic molecular subtypes of breast cancer. Breast.

[22] Goldhirsch A., Winer E.P., Coates A.S., Gelber R.D., Piccart-Gebhart M., Thurlimann B., Senn H.J., Panel m. (2013). Personalizing the treatment of women with early breast cancer: Highlights of the St Gallen International Expert Consensus on the Primary Therapy of Early Breast Cancer 2013. Ann. Oncol..

[23] Manoochehri Khoshinani H., Afshar S., Najafi R. (2016). Hypoxia: A Double-Edged Sword in Cancer Therapy. Cancer Investig..

[24] Gonzalez-Angulo A.M., Morales-Vasquez F., Hortobagyi G.N. (2007). Overview of resistance to systemic therapy in patients with breast cancer. Adv. Exp. Med. Biol..

[25] Denko N.C. (2008). Hypoxia, HIF1 and glucose metabolism in the solid tumour. Nat. Rev. Cancer.

[26] Pisco A.O., Huang S. (2015). Non-genetic cancer cell plasticity and therapy-induced stemness in tumour relapse: ‘What does not kill me strengthens me’. Br. J. Cancer.

[27] Petitjean A., Achatz M.I., Borresen-Dale A.L., Hainaut P., Olivier M. (2007). TP53 mutations in human cancers: Functional selection and impact on cancer prognosis and outcomes. Oncogene.

[28] Brosh R., Rotter V. (2009). When mutants gain new powers: News from the mutant p53 field. Nat. Rev. Cancer.

[29] Waks A.G., Winer E.P. (2019). Breast Cancer Treatment: A Review. JAMA.

